# Case Report: Efgartigimod in refractory non-tumor Lambert-Eaton myasthenic syndrome

**DOI:** 10.3389/fimmu.2026.1954344

**Published:** 2026-09-28

**Authors:** Ana Baltasar-Corral, Nuria García-Barragán, Beatriz Zarza-Sanz, Francisco Javier Buisán-Catevilla, Beatriz Montero-Llorente, Miguel Angel Rodríguez-Sagrado, Jaime Masjuan-Vallejo, Rodrigo Álvarez-Velasco

**Affiliations:** 1 Neurology Department, Hospital Universitario Ramón y Cajal, Madrid, Spain; 2 Instituto Ramón y Cajal de Investigación Sanitaria - IRYCIS, Madrid, Spain; 3 Pharmacy Department, Hospital Universitario Ramón y Cajal, Madrid, Spain

**Keywords:** Eaton-Lambert syndrome, efgartigimod, FcRn antagonist, Lambert Eaton myasthenic syndrome, new approaches and treatments

## Abstract

Lambert-Eaton myasthenic syndrome (LEMS) is a rare autoimmune disorder, associated with neoplastic disease in 50-60% of cases. In severe cases, currently available immunomodulatory therapies frequently fail to achieve adequate control. We report a 20-year-old woman with non-tumor LEMS refractory to 3,4-diaminopyridine, sequential immunosuppression with prednisone, azathioprine, mycophenolate mofetil and rituximab and plasma exchange, who remained severely disabled after two years of progressive disease. Off-label efgartigimod (1000mg subcutaneously weekly for four weeks, with the interval between cycles initially adjusted according to symptom recurrence), produced rapid and sustained clinical improvement, including recovery from a respiratory decompensation episode. The Myasthenia Gravis Activities of Daily Living (MG-ADL) score decreased from a baseline of 5–9 to a minimum of 2, and the Quantitative Myasthenia Gravis (QMG) score improved from 13–18 to 8-11. Symptoms consistently recurred toward the end of the third week of each treatment-free interval, prompting a fixed two-week interval, which stabilized clinical response and reduced fluctuation severity. This is, to our knowledge, the first report of sustained clinical benefit from FcRn inhibition in non-paraneoplastic, long-standing refractory LEMS. As in myasthenia gravis, LEMS is mediated by pathogenic circulating IgG antibodies, and these findings support FcRn inhibition as a rational treatment strategy in refractory LEMS regardless of tumor association.

## Introduction

Lambert-Eaton myasthenic syndrome (LEMS) is a rare autoimmune disorder, characterized by proximal muscle weakness, autonomic dysfunction and areflexia (1), caused by antibodies targeting P/Q-type voltage-gated calcium channels (VGCC). Paraneoplastic in 50-60% of cases, management becomes tougher in primary autoimmune cases. Its prognosis is largely determined by the presence of underlying malignancy. Despite preserved life expectancy in nontumor LEMS (NT-LEMS), long-term morbidity remains significant. Current treatment strategies rely on symptomatic agents such as 3,4-diaminopyridine and conventional immunosuppressive therapies. However, a subset of patients remains refractory, highlighting the need for targeted therapies addressing the underlying IgG-mediated pathophysiology (2).

To the best of our knowledge, we present the first published primary NT-LEMS treated successfully with efgartigimod after years of disease evolution and insufficient control with classical immunosuppressive drugs.

## Case description

A 20-year-old woman presented with months of progressive lower limb weakness, manifesting as inability to run or climb stairs, followed by involvement of the proximal upper limbs. Symptoms fluctuated and worsened with sustained activity. Neurological examination demonstrated proximal limb-girdle weakness, predominantly affecting the lower extremities and neck flexors, with fatigability and reduced deep tendon reflexes. Mild asymmetric ptosis without diplopia was noted.

The first neurophysiological study was interpreted as compatible with a postsynaptic neuromuscular junction disorder as a decremental response in compound muscle action potential amplitude (CMAP) in proximal and distal muscles was seen with both low and high-frequency repetitive stimulation. Serum testing for myasthenia gravis (MG) associated antibodies, including anti-striated muscle, anti-acetylcholine receptor and anti-muscle-specific kinase antibodies, was negative first by ELISA and then confirmed by RIA. Treatment with pyridostigmine and prednisone 20mg/day showed mild but inconsistent improvement. Higher corticosteroid doses were not tolerated because of side effects, such as insomnia, weight gain and mood swings. The picture worsened, with the patient being unable to perform basic activities such as washing hair or walking 100 meters unaided. Distal strength was also diminished. Deep tendon reflexes became absent with post-exercise facilitation prompting a second neurophysiological study. This demonstrated markedly reduced baseline CMAP amplitudes in multiple motor nerves, a decremental response at low-frequency repetitive nerve stimulation, and a 100% increment following brief maximal voluntary contraction, highly suggestive of a presynaptic neuromuscular junction disorder (**Figure 1**). It was further confirmed by high frequency repetitive stimulation. Serum antibodies against P/Q-type VGCC were subsequently found to be positive by RIA, confirming the diagnosis of LEMS two years after symptom onset.

**Figure 1:**
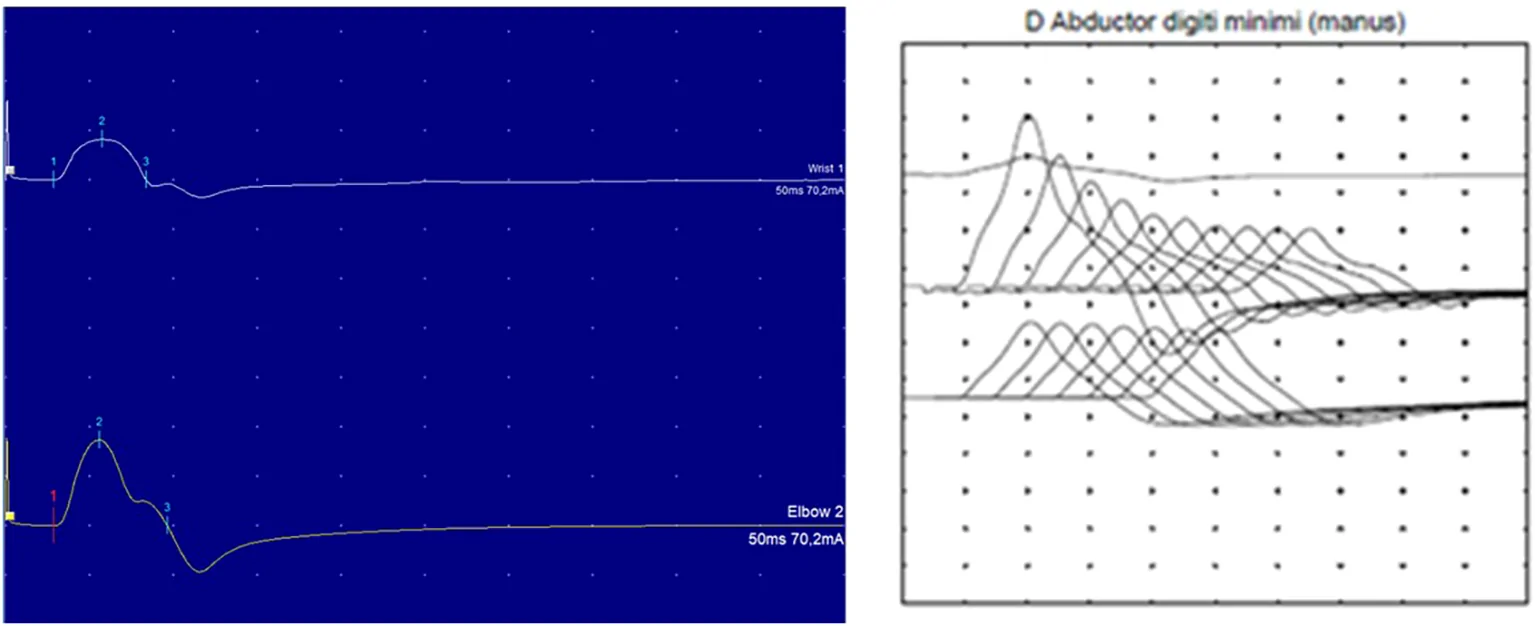
Example of the second neurophysiological study. Values of NCS and RNS. Baseline and post- exercise motor nerve conduction study of the ulnar nerve recorded from the right abductor digiti minimi muscle, with a 112% increment of the compound muscle action potential after brief maximal voluntary contraction. Decremental response after 3HZ low-frequency repetitive nerve stimulation and posterior increment following brief maximal voluntary contraction.

A body PET-CT scan was performed without neoplasms and a DELTA-P score of 0 was calculated (3). She has undergone regular tumor surveillance, being consistently normal, alternating both whole-body CT and body PET-CT scan every six months for the first two years since diagnosis. Symptomatic treatment with 3,4-diaminopyridine was initiated and gradually up-titrated, leading to marked clinical improvement but transient therapeutic effect with day-to-day symptom fluctuations.

Multiple immunosuppressive therapies were subsequently administered, including azathioprine (up to 150 mg/day) for 18 months, mycophenolate mofetil (up to 500 mg twice daily) for 11 months, and rituximab following a 4 + 2 regimen (375 mg/m² per infusion), without sustained clinical benefit and with progressive functional decline. Five sessions of plasma exchange were performed without significant improvement (**Figure 2**).

**Figure 2:**
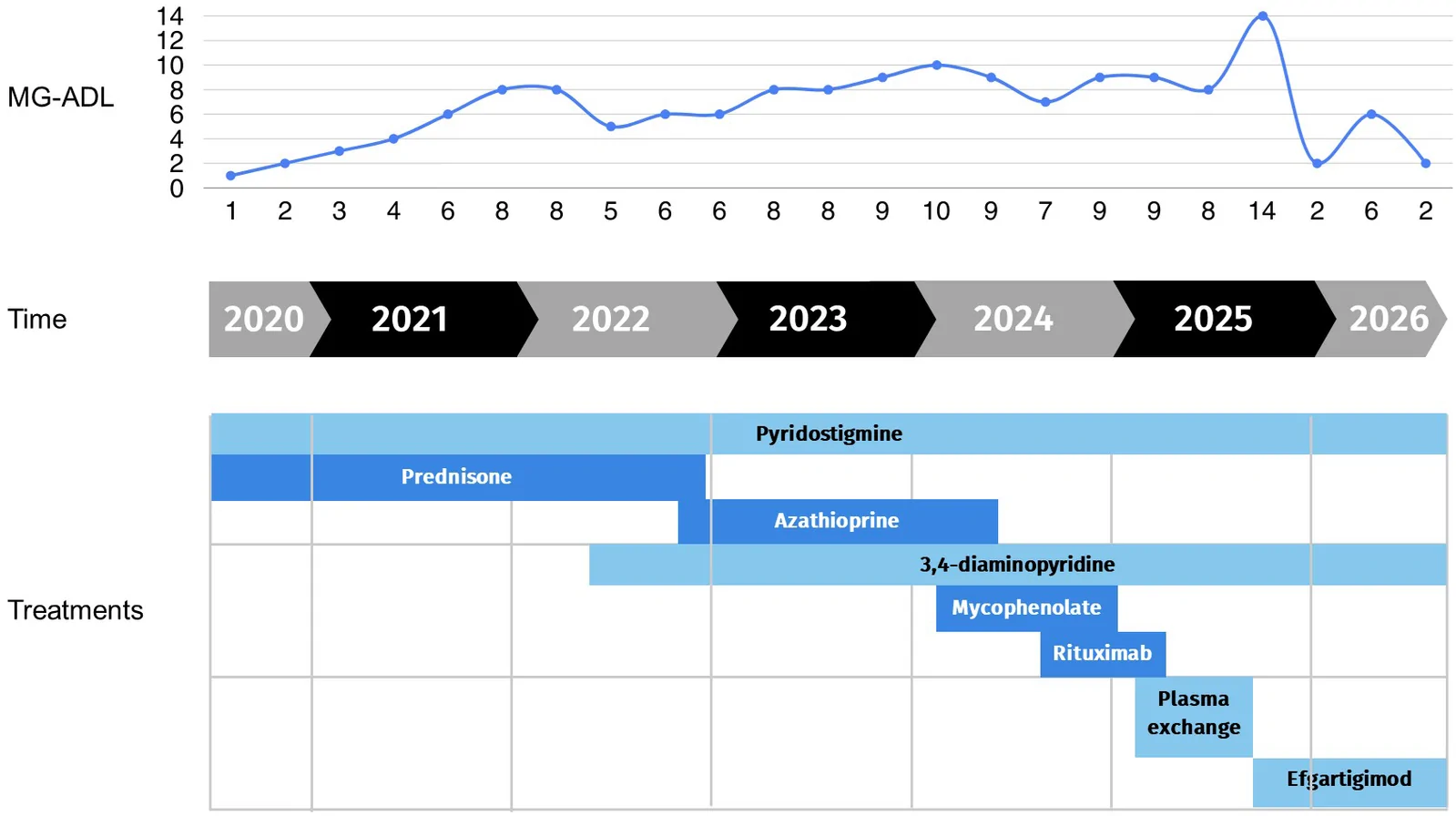
Timeline of treatments, with moderate to no response up to efgartigimod initiation.

Despite these interventions, the patient remained severely limited, unable to walk more than 50 meters or climb stairs without assistance and requiring help with several activities of daily living during periods of maximal weakness. At that time, the Myasthenia Gravis Activities of Daily Living (MG-ADL) score fluctuated between 5 and 9, and the Quantitative Myasthenia Gravis (QMG) score ranged from 13 to 18. Due to the absence of validated clinical or physical examination scales for LEMS, we elected to track the patient’s progress using widely recognized MG rating scales, supplemented by patient-reported symptoms and daily functional impairments. The QMG scale offers the advantage of capturing fatigability, which is a hallmark feature of neuromuscular junction disorders, as well as ocular and bulbar symptoms, both of which were present in our patient.

An application for off-label use of efgartigimod was submitted to the institutional pharmacy committee and granted approval.

In July 2025, the first cycle of subcutaneous efgartigimod (1000mg weekly for four weeks, as per MG protocol of treatment (4)) was administered resulting in mild clinical improvement. The patient reported longer walking distance and improved ability to perform daily activities such as washing her hair.

In September 2025, prior to the next cycle, the patient needed urgent admission because of clinical worsening requiring non-invasive mechanical ventilation (NIMV). As a trigger an infectious colitis was detected and treated empirically with Ceftriaxone 1gr/day for 7 days, without identification of a causative microorganism. Efgartigimod was reinitiated during admission, leading to rapid clinical improvement and successful weaning from NIMV within three days from the first dose.

Following the initial four-week induction cycle (July 2025) and reinitiation during the September 2025 admission, the first five cycles followed a symptom-guided protocol, with dosing intervals individually adjusted according to clinical response. A fixed two-week interval was subsequently adopted from February 2026, followed by a further shortening to a fixed weekly schedule from May 2026 onward. Symptom’s monitoring has been performed using weekly self-administered MG-ADL assessments through a validated smartphone application (5) (**Figure 3**). The MG-ADL score decreased to a minimum of 2 one week after the first dose and remained improved for several weeks after the end of each cycle. QMG score also improved from 13–18 to 8-11, with weakness of proximal limbs and neck flexors as the most improved items. Currently, she walks unaided for more than 200 meters and has regained the ability to perform complex self-care activities, such as washing hair, independently.

**Figure 3:**
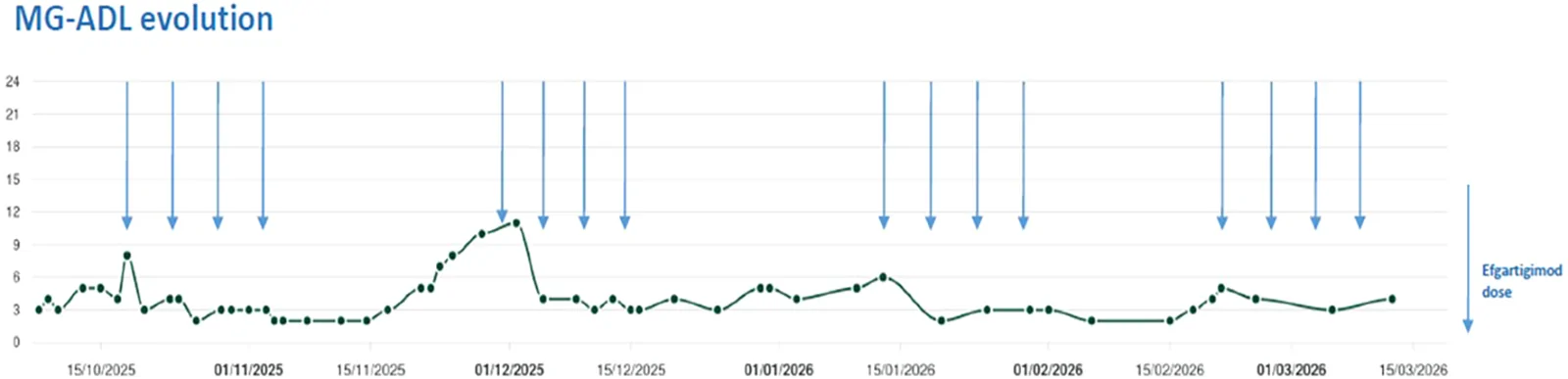
Evolution of MG-ADL reported by patient via myMGverse app, ongoing use since October 2025. The blue arrows represent every efgartigimod dose (weekly dose for four weeks).

Concomitant 3,4-diaminopyridine and pyridostigmine were maintained unchanged throughout efgartigimod treatment, while prior immunosuppressive therapies (azathioprine, mycophenolate mofetil, rituximab) remained discontinued (as seen in **Figure 2**).

## Discussion

At the time treatment was initiated, no published data were available regarding the use of efgartigimod in LEMS, to the best of our knowledge. Nevertheless, its use was considered biologically plausible. LEMS is an IgG-mediated autoimmune disorder characterized by antibodies directed against P/Q-type VGCC. Efgartigimod, a recombinant human IgG1 Fc fragment, selectively inhibits neonatal Fc receptor (FcRn)–mediated IgG recycling, thereby accelerating the degradation of pathogenic IgG autoantibodies and reducing total circulating IgG levels (6, 7). This mechanism has demonstrated clinical efficacy in other IgG-mediated disorders, including MG (4) and chronic inflammatory demyelinating polyneuropathy (6).

Given the clear antibody-mediated pathophysiology of LEMS and our patient’s refractoriness to conventional immunosuppressive therapies, FcRn blockade represented a rational targeted therapeutic strategy (7). In contrast to broad immunosuppressive approaches, efgartigimod offers selective IgG reduction with a comparatively favorable safety profile, most commonly associated with mild infections, headache, and gastrointestinal symptoms (4, 6), and without the cumulative toxicities associated with cytotoxic agents (7).

Although plasma exchange reduces circulating pathogenic antibodies, its effect is confined to the intravascular compartment. FcRn antagonists, in contrast, interrupt the IgG recycling process simultaneously across both intravascular and extravascular compartments (6), which may account for the discordant efficacy observed in our patient between these two treatments.

Regarding treatment with IVIG, it was considered but not administered: given the significant and rapid clinical deterioration at that time, plasma exchange was preferred due to its faster expected onset of action. Following the lack of response to plasma exchange, IVIG was not subsequently reconsidered. Efgartigimod was ultimately selected because of its distinct, targeted mechanism — direct FcRn-mediated IgG catabolism — its established efficacy and safety profile in myasthenia gravis (4), a pathophysiologically related antibody-mediated neuromuscular junction disorder, and the absence of a clear alternative mechanism-based option after failure of both cellular immunosuppression and antibody removal (6, 7).

Since initiation of treatment in our patient, two reports from two Chinese groups have described the successful use of efgartigimod in paraneoplastic LEMS (8, 9). Both cases demonstrated clinical improvement accompanied by reductions in antibody titers. As in our case, Zhang et al. reported rapid respiratory symptoms response after efgartigimod treatment (8). To our knowledge, this is the first report of sustained clinical benefit in non-paraneoplastic LEMS, and notably, in the setting of long-standing, refractory disease.

An additional relevant aspect of this case is the optimization of treatment interval. Initial cyclical administration resulted in clear improvement during the first two weeks following the administration, followed by predictable clinical deterioration toward the end of the third week. Shortening the treatment-free interval resulted in stabilization of symptom control and reduced fluctuation severity. This observation is consistent with evolving treatment paradigms in FcRn inhibition, in which individualized, response-guided regimens are increasingly being replaced by fixed monthly dosing cycles, particularly in patients with fluctuating symptoms.

This shortening has been welcomed by our patient, reporting less symptoms fluctuations and leading to an overall wellness she had not achieved with any prior treatment. The subcutaneous dosing allows for independent at-home treatment administration and the self-administered MG-ADL assessments through a validated smartphone application (5) helped both patient and clinicians identify and correct these fluctuations. Should symptomatic control fluctuate further or dependency on efgartigimod persist or increase, the introduction of an upstream immunosuppressant—cyclosporine being the leading candidate—targeting the antibody-producing pathway will be reconsidered as part of long-term disease management.

A serological confirmation of pathogenic antibody reduction would strengthen the causal link between efgartigimod and clinical improvement seen in our patient. P/Q-type VGCC testing by RIA is not available at our center and must be sent to a reference laboratory in another city, requiring inter-institutional approval that has been delayed. A follow-up sample has recently been approved and sent.

Prior to initiation of efgartigimod in May 2025, a blood sample was drawn to test IgG levels, with normal IgG levels (1300mg/dL, normal range: 700-1600mg/dl), as well as IgM (76.40mg/dL, normal range: 40-230) and IgA (180mg/dl, normal range: 70-400mg/dl). In October 2025, after the first two cycles, IgG levels had decreased to 560mg/dL, and continued to be reduced in February 2026 (479mg/dl) prior to the adjustment of the cycle interval. No changes were seen in IgM and IgA (October, 68.80mg/dl and 230.30mg/dl, and February, 56.40mg/dL and 208.70mg/dl, respectively).

This case supports the rationale for FcRn inhibition in refractory LEMS, including long-lasting non-paraneoplastic disease. Importantly, meaningful clinical improvement was achieved despite several years of progressive disability, indicating that chronic disease duration should not preclude consideration of targeted immunomodulatory therapy.

## Data Availability

The raw data supporting the conclusions of this article will be made available by the authors, without undue reservation.
